# Neural Substrates of Motor and Non-Motor Symptoms in Parkinson’s Disease: A Resting fMRI Study

**DOI:** 10.1371/journal.pone.0125455

**Published:** 2015-04-24

**Authors:** Kwangsun Yoo, Sun Ju Chung, Ho Sung Kim, Oh-hyeon Choung, Young-Beom Lee, Mi-Jung Kim, Sooyeoun You, Yong Jeong

**Affiliations:** 1 Laboratory for Cognitive Neuroscience and NeuroImaging, Department of Bio and Brain Engineering, KAIST, Daejeon, Republic of Korea; 2 Department of Neurology, Asan Medical Center, University of Ulsan College of Medicine, Seoul, Republic of Korea; 3 Department of Radiology, Asan Medical Center, University of Ulsan College of Medicine, Seoul, Republic of Korea; University of Michigan, UNITED STATES

## Abstract

**Background:**

Recently, non-motor symptoms of Parkinson’s disease (PD) have been considered crucial factors in determining a patient’s quality of life and have been proposed as the predominant features of the premotor phase. Researchers have investigated the relationship between non-motor symptoms and the motor laterality; however, this relationship remains disputed. This study investigated the neural connectivity correlates of non-motor and motor symptoms of PD with respect to motor laterality.

**Methods:**

Eight-seven patients with PD were recruited and classified into left-more-affected PD (n = 44) and right-more affected PD (n = 37) based on their MDS-UPDRS (Movement Disorder Society-sponsored revision of the Unified Parkinson’s Disease Rating Scale) motor examination scores. The patients underwent MRI scanning, which included resting fMRI. Brain regions were labeled as ipsilateral and contralateral to the more-affected body side. Correlation analysis between the functional connectivity across brain regions and the scores of various symptoms was performed to identify the neural connectivity correlates of each symptom.

**Results:**

The resting functional connectivity centered on the ipsilateral inferior orbito-frontal area was negatively correlated with the severity of non-motor symptoms, and the connectivity of the contralateral inferior parietal area was positively correlated with the severity of motor symptoms (p < 0.001, |r| > 0.3).

**Conclusions:**

These results suggest that the inferior orbito-frontal area may play a crucial role in non-motor dysfunctions, and that the connectivity information may be utilized as a neuroimaging biomarker for the early diagnosis of PD.

## Introduction

Motor impairments are prominent in Parkinson’s disease (PD); however non-motor symptoms have received increasingly greater attention as the features of the premotor phase that precede the motor symptoms [1]. In general, motor symptoms initiate one side of the body and progress to the opposite side and the axial muscles as the disease progresses [2]. Many clinical studies that examined motor laterality have focused on identifying a relationship between the more-affected side and other features, such as handedness or the manifestation of non-motor symptoms [3, 4]. However, it remains unclear how motor laterality is related to non-motor symptoms. Thus, it may be essential to consider the lateralized brain changes in combination with the asymmetrical motor symptoms in the study of non-motor symptoms of PD.

Resting fMRI has been widely used to investigate the large scale brain network and has been applied to patients with various brain disorders. Many studies have demonstrated a disruption in resting brain networks in neuropsychiatric disorders, including Alzheimer’s disease [5]. In the case of PD, studies have reported alterations in the functional connectivity in the default mode network and the subcortical and/or cortical motor network [6, 7]. However, to our knowledge, no study has filled the gap between lateralized connectivity changes and clinical features other than motor symptoms. In this study, we investigated the effects of motor laterality on various symptoms in terms of functional connectivity.

## Materials and Methods

### Participants

Eighty-seven consecutive PD patients were recruited in the Department of Neurology at Asan Medical Center in Seoul, Korea between March 2010 and June 2012. The diagnosis of PD was made by experienced neurologists (S.J.C., M.J.K., and S.Y.) using the United Kingdom Parkinson’s Disease Society Brain Bank clinical diagnostic criteria [8] and the mean disease duration of the whole PD patients was 4.0 (±4.1) years. Every participant underwent MR scans, clinical interviews, and neurological examinations, including the MDS-UPDRS (Movement Disorder Society-sponsored revision of the Unified Parkinson’s Disease Rating Scale) [9]. MDS-UPDRS and fMRI were obtained during practically off period. The practically off period was defined as the condition of the patients after withholding anti-parkinsonian medications for at least 12 hours. Patients with PD were divided into two groups: one consisted of patients who had dominant motor symptoms on their left body side (left-more-affected, LPD), and the other included patients with such symptoms on their right body side (right-more-affected, RPD) based on the score of the MDS-UPDRS part III (motor examination). In detail, if the difference between the summed scores of items for the limbs on each side exceeds 2 points, we defined the patient as having lateralized motor symptoms and categorized the patient as RPD (when sum of right limbs > left) or LPD (right < left). The patients with less than a 2-point difference were assumed to be symmetric and were excluded from further analysis. Six patients were included in this symmetric group. Finally we studied 44 LPD and 37 RPD patients. These 2 groups were not different in the MDS-UPDRS total score, the MDS-UPDRS part III score, the difference of summed scores between body sides on the MDS-UPDRS part III, the Hoehn and Yahr stage or their age [2]. Demographic and clinical information are presented in Table 1. The participants gave written informed consent to participate in this study. This study was approved by the Institutional Review Board of Asan Medical Center, Seoul, Korea.

**Table 1:** Demographic and clinical information of two Parkinson’s disease sub-groups.

	Left-more-affected PD	Right-more-affected PD
Subjects (sex)	44 (24F)	37 (23F)
Age	64.4 (±9.3)	62.2 (±12.7)
MDS-UPDRS	46.9 (±18.5)	48.2 (±20.0)
MDS-UPDRS I	5.6 (±.4.8)	6.0 (±4.3)
MDS-UPDRS II	11.1 (±5.8)	12.4 (±6.0)
MDS-UPDRS III	29.6 (±11.9)	29.6 (±11.9)
Motor laterality^a^	6.5 (±3.5)	7.5 (±3.5)
S&E ADL	79.8 (±6.2)	79.1 (±9.9)
H&Y stages	2.1 (±0.6)	2.1 (±0.7)

A total of 81 subjects with Parkinson’s disease were included in the functional connectivity analysis. Means (standard deviation) for each item are described.

^a^Motor laterality: a difference between the summed scores of items for limbs on each side from UPDRS part III.

PD: Parkinson’s disease; MDS-UPDRS: Movement Disorder Society-sponsored revision of the Unified Parkinson’s Disease Rating Scale; S&E ADL: Schwab & England activities of daily living; H&Y: Hoehn and Yahr.

### Acquisition of brain MRI data

Each subject underwent structural and functional MRI scanning. We performed fMRI scanning during the daytime when patients’ anti-parkinsonian medications were withheld for at least 12 hours. Therefore, fMRI was acquired in different time periods (morning, afternoon, or evening) in each patient. Data were acquired using a 3 Tesla Intera Achieva MR imaging scanner (Philips Healthcare, Best, The Netherlands). High resolution T1-weighted anatomical MRI data were obtained with the following parameters: voxel size = 0.5 x 0.5 x 0.5 mm^3^; TR = 9.9 ms; TE = 4.6 ms; flip angle = 8°; FOV (FH, AP, RL) = 240 x 240 x 180 mm^2^; matrix = 480 x 480; and 360 slices (slice orientation = sagittal). T2*-weighted MRI data (resting state fMRI) were obtained using a gradient echo planar imaging pulse sequence with the following parameters: voxel size (RL, AP, FH) = 1.72 x 1.72 x 4 mm^3^; TR = 3000 ms; TE = 35 ms; flip angle = 90°; FOV (RL, AP, FH) = 220 x 220 x 140 mm^2^; matrix = 128 x 128; and 35 slices (slice orientation = transverse). Because the resting state fMRI data were acquired in 300 seconds, the volume of one resting state fMRI was 100.

### Pre-processing of resting state fMRI

We discarded the first two volumes of fMRI for signal stabilization prior to pre-processing. We then performed pre-processing for the resting state fMRI including slice-timing correction, realignment for motion correction, co-registration, spatial normalization (into the MNI template), and smoothing (using an isotropic Gaussian kernel of 6 mm full-width at half maximum (FWHM)). The slice timing correction and spatial realignment were performed for 98 fMRI volumes. Then, the slice timing corrected and realigned fMRI data were co-registered with structural T1-weighted MRI. T1-weighted MRI was used as a source image of normalization to register fMRI data in the Montreal Neurological Institute (MNI) template. The transformation matrix from T1-weighted MRI to the MNI-152 T1 stereotactic template was first calculated and then applied to co-registered fMRI data. Finally, to increase the signal to noise ratio, normalized fMRI data were smoothed using an isotropic Gaussian kernel of 6 mm FWHM. All pre-processing steps were performed using Statistical Parametric Mapping 8 in MATLAB R2011b (7.13) (Natick, MA, USA).

### Correlation analysis between intrinsic functional connectivity and clinical features

We employed 116 automated anatomical labeling (AAL) region of interests (ROIs) that were originally labelled as the left or right hemispheric region [10]. In this study, we re-categorized these ROIs as located in the ipsilateral hemisphere (left hemisphere for LPD and right hemisphere for RPD) or contralateral hemisphere (right hemisphere for LPD and left hemisphere for RPD) in accordance with the body side of the dominant motor symptoms. We extracted the averaged time-series for each ROI from the individual normalized fMRI data. Then, as a measure of functional connectivity, we calculated a Pearson’s correlation coefficient for each pair of the 116 time-series. Finally, we performed correlation analyses between functional connectivity and clinical features. We estimated a Pearson’s correlation coefficient between functional connectivity calculated above and MDS-UPDRS scores. The correlation was taken significant when the absolute value of correlation coefficient ‘r’ is over 0.3 and p-value of correlation is below 0.001, (|r| > 0.3 & p < 0.001). For clinical features, we used the scores of the MDS-UPDRS parts I, II, and III and the Schwab and England (S&E) activities of daily living (ADL), as previously described.

## Results

### Neural connectivity substrates for non-motor symptoms and signs

#### MDS-UPDRS part I (Non-motor aspects of experiences of daily living)

**Fig 1:**
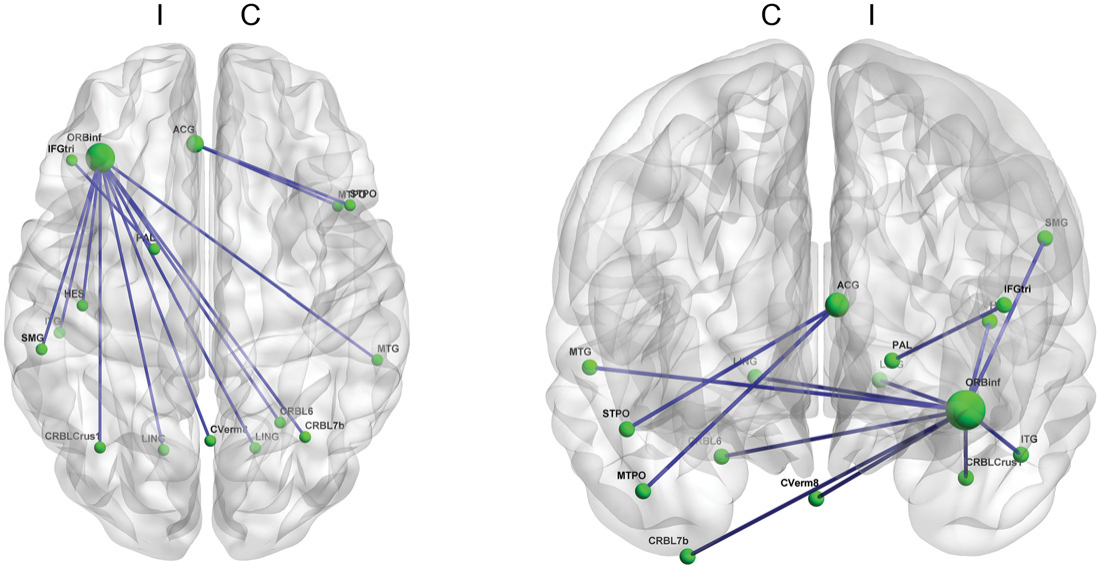
Functional connectivity correlated with the MDS-UPDRS part I score. The inferior orbito-frontal area in the ipsilateral hemisphere has substantial functional connectivity negatively correlated with the severity of non-motor symptoms. Other ipsilateral inferior frontal areas such as the pars triangularis and the anterior cingulate cortex also exhibit functional connectivity correlated with the severity of non-motor symptoms. (p<0.001) (Blue line: a functional connectivity negatively correlated with the MDS-UPDRS part I score; C: contralateral hemisphere & I: ipsilateral hemisphere; MDS-UPDRS: Movement Disorder Society-sponsored revision of the Unified Parkinson’s Disease Rating Scale)

We identified significant negative correlations between the MDS-UPDRS part I score and functional connectivity in brain areas centered at the ipsilateral inferior frontal areas and the anterior cingulate gyrus (p < 0.001, Fig 1 and S1 Table). The identified functional connectivity of frontal areas was with cerebellum, globus pallidus, and parietal and temporal cortices.

### Neural connectivity substrates for motor symptoms and signs

#### MDS-UPDRS part II (Motor aspects of experiences of daily living)

**Fig 2:**
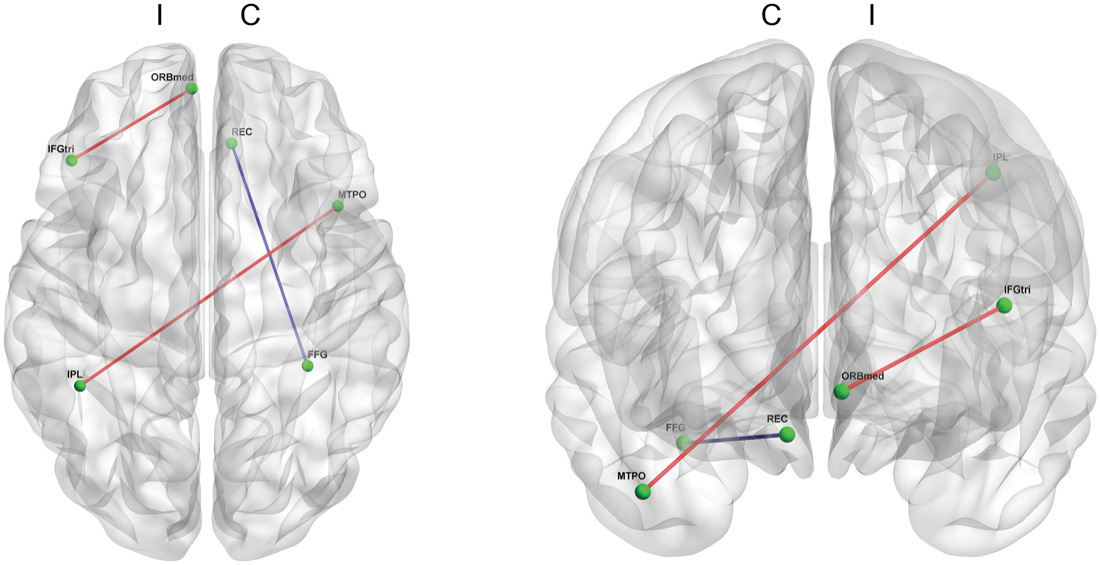
Functional connectivity correlated with the MDS-UPDRS part II score. Limited functional connectivity is shown to correlate with the MDS-UPDRS part II score. This score positively correlates with the functional connectivity between the pars triangularis and the orbital part of the medial frontal gyrus within the ipsilateral hemisphere, and with the connectivity between ipsilateral inferior parietal lobule and contralateral middle temporal pole. The MDS-UPDRS part II has a negative correlation with the connectivity between rectal gyrus and the fusiform gyrus in the contralateral hemisphere. (p<0.001) (Blue line: a functional connectivity negatively correlated with the MDS-UPDRS part II score & red line: a functional connectivity positively correlated with the MDS-UPDRS part II score; C: contralateral hemisphere & I: ipsilateral hemisphere; MDS-UPDRS: Movement Disorder Society-sponsored revision of the Unified Parkinson’s Disease Rating Scale)

For the MDS-UPDRS part II, the score had significant correlations with several functional connectivities (p < 0.001, Fig 2). The part II score had positive correlations with connectivity between the ipsilateral pars triangularis and the orbital part of the medial frontal gyrus, and with connectivity between the ipsilateral inferior parietal lobule and the contralateral middle temporal pole. The part II score also had a negative correlation with connectivity between the rectal gyrus and the fusiform gyrus in the contralateral hemisphere.

#### MDS-UPDRS part III (Motor examination)

**Fig 3:**
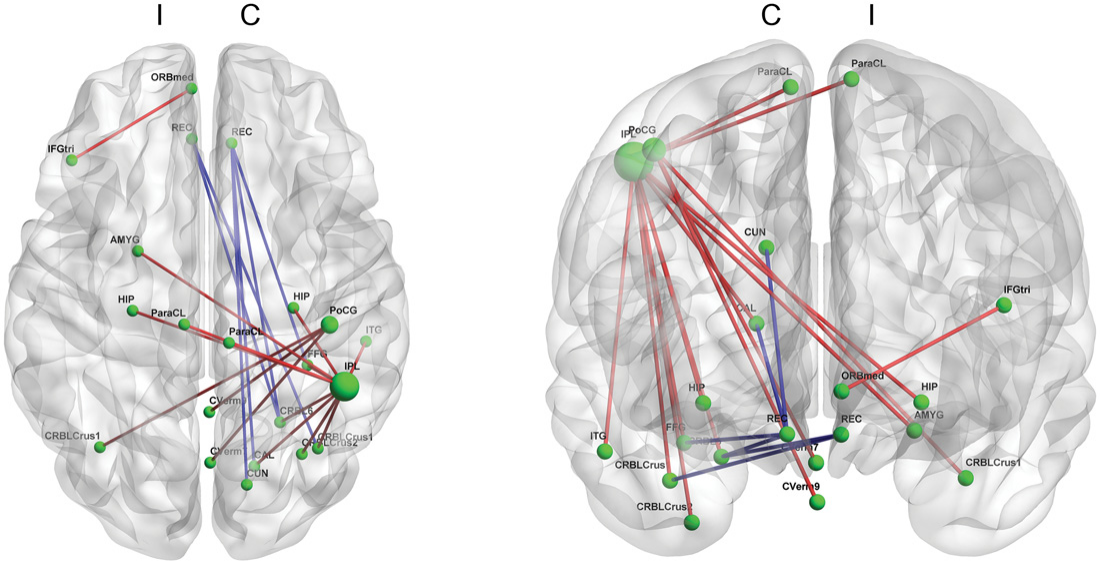
Functional connectivity correlated with the MDS-UPDRS part III score. The inferior parietal area in the contralateral hemisphere has substantial functional connectivity positively correlated with the severity of motor symptoms. The contralateral postcentral gyrus also exhibits functional connectivity positively correlated with the severity of motor symptoms. Connectivity between the pars triangularis and the orbital part of the medial frontal gyrus is also positively correlated with the MDS-UPDRS part III score, whereas the connectivity of bilateral rectus gyri is negatively correlated with this score. (p<0.001) (Blue line: a functional connectivity negatively correlated with the MDS-UPDRS part III score & red line: a functional connectivity positively correlated with the MDS-UPDRS part III score; C: contralateral hemisphere & I: ipsilateral hemisphere; MDS-UPDRS: Movement Disorder Society-sponsored revision of the Unified Parkinson’s Disease Rating Scale)

For the MDS-UPDRS part III, the score had positive correlations with functional connectivity of the contralateral parietal areas, but had negative correlations with connectivity of bilateral rectal gyri (p < 0.001, Fig 3 and S2 Table). Specifically, the functional connectivity of the contralateral inferior parietal area with other brain regions, including the contralateral cerebellum and the bilateral hippocampi and paracentral lobule, and the connectivity of the contralateral postcentral gyrus with the cerebellum were positively correlated with the score. The functional connectivity of the ipsilateral rectal gyrus with the contralateral cerebellar regions and the connectivity of the contralateral rectal gyrus with other brain regions were negatively correlated with the score.

### Neural connectivity substrates for S&E ADL

**Fig 4:**
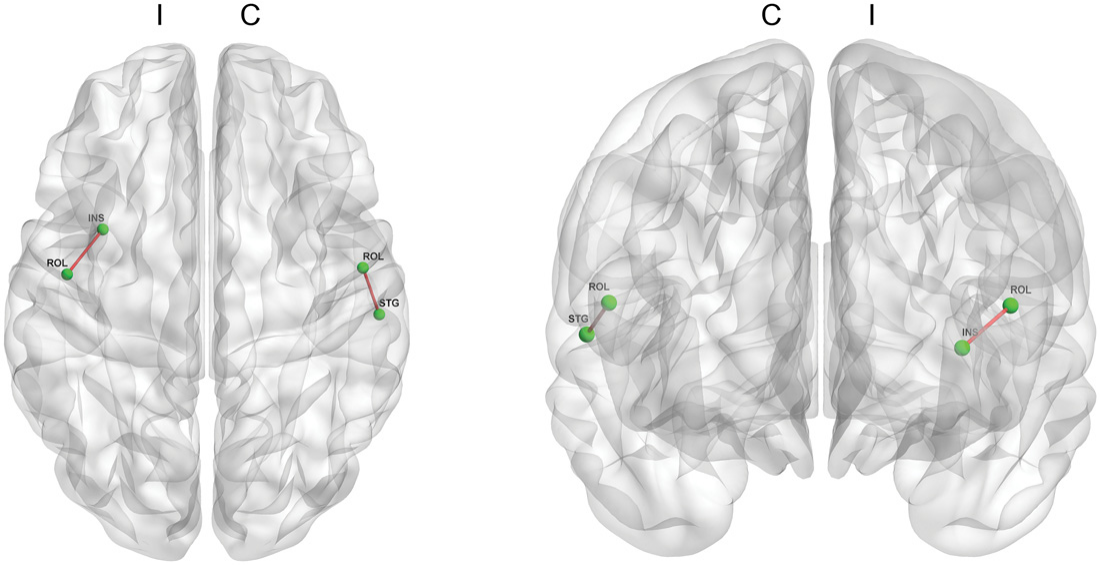
Functional connectivity correlated with the S&E ADL score. The functional connectivity between the Rolandic operculum and the insula in the ipsilateral hemisphere and between the Rolandic operculum and the superior temporal gyrus in the contralateral hemisphere are positively correlated with the S&E ADL score. (p<0.001) (Red line: a functional connectivity positively correlated with the S&E ADL score; C: contralateral hemisphere & I: ipsilateral hemisphere; S&E ADL: Schwab and England activities of daily living).

Finally, we identified a correlation between the functional connectivity and the S&E ADL score (p < 0.001, Fig 4). The functional connectivities between the ipsilateral Rolandic operculum and the insula and between the contralateral Rolandic operculum and the superior temporal gyrus were positively correlated with score of S&E ADL score.

## Discussion

In this study, we investigated the neural connectivity substrates of non-motor and motor impairments in PD with regard to the laterality of motor symptoms and signs. To accomplish this task, we acquired resting state fMRI data from patients with PD and analyzed the functional connectivity profile after separating brain hemispheres into the ipsilateral and contralateral sides with respect to the more-affected body side according to the UPDRS part III score. Then, we measured the correlation of functional connectivity with the scores of MDS-UPDRS parts I, II and III and S&E ADL. We determined that the resting functional connectivity of the inferior orbito-frontal area and the anterior cingulate cortex in the ipsilateral hemisphere evidently reflect the severity of non-motor symptoms in patients with PD. We also revealed that the functional connectivity of the contralateral inferior parietal areas represents the deterioration of motor functions.

The laterality of motor symptoms and signs is a well-known feature in patients with PD [11]. Typically, motor symptoms present with motor dysfunction on one side and then progress to the opposite side [2]. The onset side generally exhibits worse symptoms and signs. Previous brain imaging studies have shown that this laterality of motor symptoms is related to asymmetric brain changes in the striatum and the substantia nigra [12, 13]. Clinical studies have investigated the relationship between the motor laterality and non-motor manifestations. However, these studies have reported inconsistent results; poor cognition in left-onset PD [14], the relation between right-side symptoms and cognition [15], or no differences between the LPD and RPD [16]; greater anxiety and depression in LPD [17] or severe psychosis in extreme RPD [4]; and no consistent relation between motor laterality and non-motor symptoms other than psychosis [4]. These findings suggest that lateralized brain changes must be considered in the study of non-motor symptoms. However, no research, to date, has studied the relationship between asymmetric brain changes and non-motor symptoms.

Regarding the non-motor symptoms, the functional connectivity of the inferior orbito-frontal area ipsilateral to the more-affected side was evidently correlated with the severity of the non-motor symptoms. The inferior orbito-frontal area is known to be involved in reward-processing and decision-making, emotion, depression and attention [18–21]. In PD, structural imaging studies have shown gray matter reductions and alterations of DTI indices (decreased fractional anisotropy and increased mean diffusivity) in the orbito-frontal area [22–24]. In addition, other imaging studies have directly shown that the orbitofrontal area is involved in cognitive impairment, hallucination, depression, apathy, and pathologic gambling in PD [25–29]. Because the items of the MDS-UPDRS part I (e.g., cognitive impairment, hallucination and psychosis, several mood disorders, and features of dopamine dysregulation syndrome) are tightly related to these brain functions, a high correlation between the connectivity of the orbitofrontal area and the severity of non-motor symptoms is plausible. Thus, the decreased connectivity of the orbitofrontal area can be assumed to be a core correlate of non-motor symptoms. In other words, this decreased frontal connectivity with severe non-motor symptoms might represent a pathophysiology of non-motor symptoms. In addition, as reported by Braak staging, the orbitofrontal area is known to be involved in the early stage of PD [30]. The involvement of the orbital cortex may be related to the accumulation of α-synuclein-related pathology in the underneath anterior olfactory nucleus or in the basal forebrain [31]. Because the non-motor symptoms such as depression, autonomic dysfunctions, and hallucination would precede the motor symptoms in PD [1, 32], the connectivity changes centered in the orbitofrontal cortex may provide an early signature of the disease.

We also demonstrated that the functional connectivity of the anterior cingulate cortex is correlated with the severity of non-motor symptoms. The anterior cingulate cortex has a well-established role in reward-processing and decision-making, apathy, attention, emotion, depression, and sympathetic activity [19–21, 33, 34]. A DTI study demonstrated an alteration of FA in the anterior cingulate cortex in PD [24]. A functional imaging study in PD reported impaired activation of the anterior cingulate cortex during a free-choice task [35]. Other studies have also identified an involvement of the anterior cingulate cortex in apathy, hallucination, and dementia in PD [25, 27, 29, 37]. Furthermore, it has been reported that Lewy body density in the anterior cingulate cortex could predict cognitive deficits in patients with PD [36]. Along with previous evidence, we carefully suggest that the functional connectivity of the inferior orbito-frontal area and anterior cingulate cortex might be able to provide an early biomarker for non-motor symptoms in the premotor or prodromal stage of PD. With respect to laterality, we are not certain why the changes were identified in the ipsilateral hemisphere to the more affected body side. We can speculate reasons for this involvement of ipsilateral connectivity. Parkinson’s pathology is known to appear first in the contralateral hemisphere [11]. So, we speculate that functional connectivity of contralateral frontal areas was already too lowered to show any correlation with scores of non-motor symptoms, while ipsilateral connectivity is also getting lowered, however remained enough to show correlation with non-motor symptoms scores.

We also demonstrated a correlation between the functional connectivity of the inferior parietal areas with MDS-UPDRS part III (motor examination) score. We expected that the connectivity correlates would be centered at primary or secondary motor cortical areas or the related basal nuclei because these areas are necessary for the final execution of motor command. On the other hand, the inferior parietal regions are involved in cognitive motor function as well as the primary function. Previous studies have shown that the inferior parietal regions are involved in many motor tasks that range from simple motions to complex movements. It is well known that the inferior parietal regions are involved in motor execution, motor-skill learning, tool-use execution [38], and simple sequence finger tapping [39]. In PD, the prominent motor dysfunction is primarily related to primary motor control; however, studies have shown that cognitive motor function is also impaired [40]. In addition, some studies have shown that PD patients scored significantly lower in testing for ideomotor apraxia, which is known to occur with inferior parietal area damage [41]. However, we did not determine whether the patients had these cognitive motor dysfunctions in this study.

One interesting finding is that the correlation between the functional connectivity of the contralateral inferior parietal area with the UPDRS part III score was positive. This finding indicates that patients with PD who display more severe motor symptoms have a higher functional connectivity. This finding is consistent with previous imaging studies that have shown that lower functional connectivity is related to higher performance. These studies reported that experts (at motor exercise) have lower resting functional connectivity and lower activation during the specific task compared with beginners [38, 42]. In the case of PD, patients show higher activation in inferior parietal area during a motor task (e.g., sequencing movement) compared with normal healthy controls [43]. It has also been shown that the functional connectivity of the parietal area is increased in PD [44]. Thus, worsening of the motor performance in PD may induce strengthening of the functional connectivity potentially as a mechanism for compensation.

This study has several limitations. First, we did not compare the functional connectivity of PD with normal healthy controls. It would be helpful to reveal asymmetrical connectivity changes occurred in PD compared with normal healthy controls with respect to the laterality of motor symptoms and signs. Second, we categorized patients with PD into RPD and LPD without considering the type of motor symptoms and signs. It has been suggested that different motor symptoms of bradykinesia, rigidity, and resting tremor are involved in distinct alterations of brain networks (e.g., the different frequency range of the brain waves obtained from EEG and MEG). However, we did not address these motor symptoms. Third, we combined items that were included in each UPDRS part. For example, the UPDRS part I contained diverse non-motor-related items, such as hallucination, constipation, dopaminergic dysregulation syndrome, and sleep problems. These features likely have their own distinct causes and neural correlates; hence, they might need to be studied separately. However, the narrow range of this individual measure hampers the correlation analysis. Forth, regarding the selection of ROIs, we employed AAL ROIs and divided the brain into 116 regions. This set of ROIs might not be appropriate because of the absence of small regions, such as the substantia nigra; however, there is no standard method for ROI selection in the analysis of the brain imaging data. Finally, the last limitation is that we performed collective analyses of the left and right brain hemispheres together. It is well established that each hemisphere is specialized for certain functions, such as language, visuospatial function and motor function. In addition, each hemisphere is not the same in terms of anatomy. Because we assumed both hemispheres were the same, there may be a possibility of misapprehension. If the function were lateralized to one hemisphere or if both hemispheres worked reciprocal inhibition ways, the results would be diluted. However, the non-motor symptoms and signs of PD that we investigated in this study are known to be relatively less lateralized; thus, this bias may have minimal influence on the results.

## Conclusions

The aim of this study was to reveal the neural connectivity substrates of non-motor symptoms with respect to the laterality of motor symptoms. We demonstrated that the functional connectivity of the ipsilateral inferior orbito-frontal gyrus and the anterior cingulate gyrus is significantly correlated with the severity of non-motor symptoms, whereas the contralateral inferior parietal area is correlated with the severity of motor dysfunction.

## Supporting Information

S1 Material Detail information on patients with Parkinson’s disease (PD). Information on patients including medications, dominant motor symptoms, types of several non-motor symptoms is described.
(DOCX)

S1 Table Functional connectivity correlated with the MDS-UPDRS part I score. Functional connectivity which is significantly correlated with the MDS-UPDRS part I score is represented (p < 0.001, |r| > 0.3). Correlation analysis was performed using Pearson’s correlation. Pearson’s correlation coefficient r is described.
(DOCX)

S2 Table Functional connectivity correlated with the MDS-UPDRS part III score. Functional connectivity which is significantly correlated with the MDS-UPDRS part III score is represented (p < 0.001, |r| > 0.3). Correlation analysis was performed using Pearson’s correlation. Pearson’s correlation coefficient r is described.
(DOCX)
