# Supplementary material for: Neural Substrates of Motor and Non-Motor Symptoms in Parkinson’s Disease: A Resting fMRI Study
Source: PLoS One. 2015 Apr 24;10(4):e0125455. doi: 10.1371/journal.pone.0125455 (PMC4409348; doi:10.1371/journal.pone.0125455)
Supplement: S1 Material — Information on patients including medications, dominant motor symptoms, types of several non-motor symptoms is described. (DOCX) [file pone.0125455.s001.docx]

**Material S1. Detail information on patients with Parkinson’s disease (PD)**

**Participants: patients with PD**

A total of 81 PD patients were included in our analysis. Regard to dominant motor symptoms, there were 7 tremor dominant and 66 with akinetic-rigid motor subtype PD patients. The remaining patients were mixed type. Seven patients met the criteria for the dementia [1]. Twenty-seven PD patients had psychotic features, 9 PD patients had depression, 7 PD patients had excessive daytime somnolence, and no PD patients had impulse control disorders.

The patients are under medications as follows;

| Medication | The number of patients treated (%) |
| --- | --- |
| levodopa | 79 (97.5%) |
| ropinirole | 14 (17.3%) |
| pramipexole | 13 (16%) |
| entacapone | 5 (6.2%) |
| selegiline | 5 (6.2%) |
| amantadine | 23 (28.4%) |

Mean levodopa equivalent dose was 506.77 ± 345.85 in right-more-affected PD patients and 552.78 ± 393.91 in left-more-affected PD patients (no significant difference, p-value 0.577). The mean dose of the whole 81 PD patients was 531.76 ± 371.16. There were 5 PD patients who showed levodopa-induced dyskinesia and 3 PD patients who had wearing-off phenomenon.

The correlation between scores of MDS-UPDRS part I, II and III are following. Pearson’s correlation coefficient was estimated in each group.

| **LPD** | Part I & II | Part I & III | Part II & III |
| --- | --- | --- | --- |
| Correlation | 0.28 | 0.29 | 0.82 |
| P-value | 0.07 | 0.05 | <0.0001 |

| **RPD** | Part I & II | Part I & III | Part II & III |
| --- | --- | --- | --- |
| Correlation | 0.57 | 0.43 | 0.87 |
| P-value | 0.0002 | 0.0073 | <0.0001 |

**Reference**

1. Emre M, Aarsland D, Brown R, Burn DJ, Duyckaerts C et al. (2007) Clinical diagnostic criteria for dementia associated with Parkinson's disease. Mov Disord 22:1689-1707
