# Supplementary material for: Neural Substrates of Motor and Non-Motor Symptoms in Parkinson’s Disease: A Resting fMRI Study
Source: PLoS One. 2015 Apr 24;10(4):e0125455. doi: 10.1371/journal.pone.0125455 (PMC4409348; doi:10.1371/journal.pone.0125455)
Supplement: S1 Table — Functional connectivity which is significantly correlated with the MDS-UPDRS part I score is represented (p < 0.001, |r| > 0.3). Correlation analysis was performed using Pearson’s correlation. Pearson’s correlation coefficient r is described. (DOCX) [file pone.0125455.s002.docx]

**Table S1. Functional connectivity correlated with the MDS-UPDRS part I score.** Functional connectivity which is significantly correlated with the MDS-UPDRS part I score is represented (p < 0.001, |r| > 0.3). Correlation analysis was performed using Pearson’s correlation. Pearson’s correlation coefficient r is described.

| Functional connectivity between | r |
| --- | --- |
| Frontal_Inf_Orb_i & Cerebellum_6_c | -0.359 |
| Frontal_Inf_Orb_i & Cerebellum_7b _c | -0.368 |
| Frontal_Inf_Orb_i & Cerebellum_Crus1_i | -0.370 |
| Frontal_Inf_Orb_i & SupraMarginal_i | -0.427 |
| Frontal_Inf_Orb_i & Heschl_i | -0.408 |
| Frontal_Inf_Orb_i & Lingual_i | -0.397 |
| Frontal_Inf_Orb_i & Lingual_c | -0.391 |
| Frontal_Inf_Orb_i & Temporal_Inf_i | -0.401 |
| Frontal_Inf_Orb_i & Temporal_Mid_c | -0.369 |
| Frontal_Inf_Orb_i & Vermis_8 | -0.363 |
| Frontal_Inf_Tri_i & Pallidum_i | -0.399 |
| Cingulum_Ant_i & Temporal_Pole_Mid_c | -0.412 |
| Cingulum_Ant_i & Temporal_Pole_Sup_c | -0.375 |

r: Pearson’s correlation coefficient (p < 0.001)

c: contralateral region & i: ipsilateral region

MDS-UPDRS: Movement Disorder Society-sponsored revision of the Unified Parkinson’s Disease Rating Scale
