# Supplementary material for: Neural Substrates of Motor and Non-Motor Symptoms in Parkinson’s Disease: A Resting fMRI Study
Source: PLoS One. 2015 Apr 24;10(4):e0125455. doi: 10.1371/journal.pone.0125455 (PMC4409348; doi:10.1371/journal.pone.0125455)
Supplement: S2 Table — Functional connectivity which is significantly correlated with the MDS-UPDRS part III score is represented (p < 0.001, |r| > 0.3). Correlation analysis was performed using Pearson’s correlation. Pearson’s correlation coefficient r is described. (DOCX) [file pone.0125455.s003.docx]

**Table S2. Functional connectivity correlated with the MDS-UPDRS part III score.** Functional connectivity which is significantly correlated with the MDS-UPDRS part III score is represented (p < 0.001, |r| > 0.3). Correlation analysis was performed using Pearson’s correlation. Pearson’s correlation coefficient r is described.

| Functional connectivity between | r |
| --- | --- |
| Parietal_Inf_c & Amygdala_i | 0.368 |
| Parietal_Inf_c & Calcarine_c | 0.392 |
| Parietal_Inf_c & Cerebellum_6_c | 0.410 |
| Parietal_Inf_c & Cerebellum_Crus1_c | 0.441 |
| Parietal_Inf_c & Cerebelum_Crus2_c | 0.362 |
| Parietal_Inf_c & Fusiform_c | 0.371 |
| Parietal_Inf_c & Hippocampus_i | 0.362 |
| Parietal_Inf_c & Hippocampus_c | 0.393 |
| Parietal_Inf_c & Paracentral_Lobule_i | 0.361 |
| Parietal_Inf_c & Paracentral_Lobule_c | 0.361 |
| Parietal_Inf_c & Temporal_Inf_c | 0.395 |
| Postcentral_c & Cerebelum_Crus1_i | 0.413 |
| Postcentral_c & Vermis_7 | 0.369 |
| Postcentral_c & Vermis_9 | 0.400 |
| Frontal_Inf_Tri_i & Frontal_Med_Orb_i | 0.390 |
| Rectus_i & Cerebellum_6_c | -0.400 |
| Rectus_i & Cerebellum_Crus1_c | -0.380 |
| Rectus_c & Calcarine_c | -0.365 |
| Rectus_c & Cerebellum_6_c | -0.381 |
| Rectus_c & Cuneus_c | -0.379 |
| Rectus_c & Fusiform_c | -0.408 |

r: Pearson’s correlation coefficient (p < 0.001)

c: contralateral region & i: ipsilateral region

MDS-UPDRS: Movement Disorder Society-sponsored revision of the Unified Parkinson’s Disease Rating Scale
